# Endometriosis Communities on Reddit: Quantitative Analysis

**DOI:** 10.2196/57987

**Published:** 2025-03-31

**Authors:** Federica Bologna, Rosamond Thalken, Kristen Pepin, Matthew Wilkens

**Affiliations:** 1 Department of Information Science Cornell University Ithaca, NY United States; 2 Department of Obstetrics and Gynecology Weill Cornell Medicine New York, NY United States

**Keywords:** online health communities, patient-centered care, chronic disease, internet, consumer health information, self-help groups, community networks, information science, social support

## Abstract

**Background:**

Endometriosis is a chronic condition that affects 10% of the people with a uterus. Due to the complex social and psychological impacts caused by this condition, people with endometriosis often turn to online health communities (OHCs) for support.

**Objective:**

Prior work identifies a lack of large-scale analyses of the experiences of patients with endometriosis and of OHCs. This study aims to fill this gap by investigating aspects of the condition and aggregate user needs that emerge from 2 endometriosis OHCs, *r/Endo* and *r/endometriosis*.

**Methods:**

We used latent Dirichlet allocation topic modeling, an unsupervised machine learning method, to extract the subject matter (“topic”) of >30,000 posts and >300,000 comments. In addition to latent Dirichlet allocation, we leveraged supervised classification. Specifically, we fine-tuned a series of the DistilBERT models to identify the people and relationships (*personas*) a post mentions as well as the type of support that the post seeks (*intent*). Combining the results of these 2 methods, we identified associations between a post’s topic, the personas mentioned, and the post’s intent.

**Results:**

The most discussed topics in posts were *medical stories*, *medical appointments*, *sharing symptoms*, *menstruation*, and *empathy*. Through the combination of the results from topic modeling and supervised classification, we found that when discussing *medical appointments*, users were more likely to mention the *endometriosis OHCs* than *medical professionals*. *Medical professional* was the least likely of any persona to be associated with *empathy*. Posts that mentioned *partner* or *family* were likely to discuss topics from the *life issues* category, particularly *fertility*. Users sought experiential knowledge regarding treatments and health care processes, and they also wished to vent and establish emotional connections about the life-altering aspects of the condition.

**Conclusions:**

We conclude that members of the OHCs need greater empathy within clinical settings, easier access to appointments, more information on care pathways, and more support for their loved ones. Endometriosis OHCs currently fulfill some of these needs as they provide members with a space where they can receive validation, discuss care pathways, and learn to manage symptoms. This study demonstrates the value of quantitative analyses of OHCs. Computational analyses can support and extend findings from small-scale studies about patient experiences and provide insights into hard-to-reach groups. Finally, we provide recommendations for clinical practice and medical training programs.

## Introduction

### Contribution

Research on the experiences of patients with endometriosis highlights several areas of endometriosis care that require improvement. Medical treatments should be more holistic, taking into consideration the social, emotional, and psychological costs of endometriosis for patients and their loved ones [1-3]. Health care providers should improve their communication to validate patients’ concerns, meet their informational needs, and avoid misunderstandings [4-6]. Because loved ones are also affected by this condition, they should receive education and training on it from health care professionals [1,7-9].

Prior work identifies a lack of large-scale mixed methods analyses on patient experiences and online communities, which could further support these findings [1,10]. Existing qualitative research on patient experiences has been limited to small patient samples. Previous quantitative analyses, though larger in scale, are limited to ontologies that are defined by researchers rather than those inferred from patient narratives [1,10,11].

This study fills this gap by providing a large-scale analysis of user behavior in 2 endometriosis online health communities (OHCs), *r/Endo* and *r/endometriosis*, on the social media platform Reddit (Reddit, Inc). Using natural language processing and qualitative analysis, we identified and mapped the associations between a post’s subject matter (“topics”), the people and relationships (“personas”) mentioned, and the type of support the post seeks (“intent”). We investigated 2 research questions (RQs):

- RQ1—What aspects of the endometriosis experience are discussed in OHCs?
- RQ2—Which aggregate unmet needs emerge from the OHCs?

Most studies on the effects of endometriosis on quality of life fail to investigate the impact of the condition on loved ones and informal caregivers as well as adolescents [1]. Instead, they only include people with an endometriosis diagnosis within the research population [5,6,12-14]. Given the long average delay between symptom onset and diagnosis [2,4], many adolescents with endometriosis are missed by this research.

Studying *r/Endo* and *r/endometriosis*, we discovered the unmet needs of these hard-to-reach groups. The endometriosis OHCs of interest in this study are open and accessible to anyone regardless of whether they have a diagnosis or not. As a result, numerous members self-identify as belonging to populations that have been missed by endometriosis research, that is, people who have yet to receive a diagnosis, adolescents, and loved ones of people with a diagnosis. Moreover, due to the pseudonymity afforded by the platforms, users feel more comfortable discussing needs that they might not have the time or courage to address in clinical settings.

Researchers have also highlighted OHCs’ role in the improvement of health care [15,16]. An existing study of a polycystic ovary syndrome (PCOS) subreddit found concordance between trends from laboratory results posted to the OHC and trends from clinical research. This indicates that, although OHCs often include patients that are typically excluded from clinical trials (such as those with multiple conditions), studying these communities is useful to understand patient populations [17]. Indeed, content analysis of these communities reveals patterns across patients’ experiences of care and symptoms [18-21], and OHC members’ expertise in providing support to peers could be leveraged to deliver health care interventions and programs [16,22-24].

### Endometriosis

Endometriosis is a chronic condition that affects 10% of people with a uterus. It is characterized by the presence of uterine lining tissue outside of the uterus [2]. This condition causes a range of painful, persistent, and life-altering symptoms, including, but not limited to, chronic pelvic pain, painful menstruation, constipation, painful urination, painful sexual intercourse, and infertility. Its estimated annual economic impact exceeds US $60 billion in direct treatment costs and lost productivity in the United States alone [25]. There is no cure for endometriosis; therefore, treatment focuses on symptom management and relief [2,26,27]. Treatments might include hormonal therapy, surgical removal of endometriosis lesions, and fertility treatment. However, such therapies cause numerous side effects and rarely provide long-term relief to patients [26].

Due to the absence of condition-specific symptoms and biomarkers, the normalization of menstrual pain, the need for surgery to make a diagnosis, and the lack of knowledge about the condition by both the public and clinicians, the average time until diagnosis is estimated to be between 6 and 11 years, depending on the health care system of reference [4,28,29]. A confirmed diagnosis can only be reached through laparoscopic excision of endometriosis tissue, an invasive surgical procedure [30].

Patients with endometriosis face numerous difficulties during their health care journeys. They not only struggle to find information but also encounter negative attitudes from physicians [5,12,31]. Patients’ concerns are often dismissed as “just period pain” by health care providers [13]. Negative attitudes seem to derive from physicians’ own discomfort with unexplained symptoms [32] as well as from the continued presence of hysteria discourse and androcentric views in medical literature [33].

Because of these interconnected factors, endometriosis has dire impacts on patients’ quality of life [34]. This condition forces people to leave their education and employment and opt out of social events and everyday activities. Due to sexual pain and infertility, patients may feel inadequate as partners and fear abandonment [1].

Patients with endometriosis require support from partners, family members, and friends to overcome these struggles and receive a diagnosis [3,29]. Self-care practices are time-consuming and labor-intensive for both patients and their loved ones. As patients focus on following complex treatment regimens and become expert in their own care [6,35-37], a wide range of other responsibilities falls onto their partners and family members. These responsibilities can include financial and housekeeping duties, helping to navigate the health care system, and relaying medical information, among others [38-41].

### Background on OHCs

OHCs are groups of individuals who come together on an internet-based platform (eg, social media, website, or forum) to discuss general or condition-specific medical topics. Members may be patients, medical professionals, informal caregivers, patients’ loved ones, or members of the general public [28,29].

OHCs have been shown to provide support to users who experience dissatisfaction or constrained access to medical care, limited social support, or the absence of a local community of people with the same condition [38,42]. Indeed, some members join OHCs after feeling alienated from the medical community or becoming distrustful of medical knowledge and care [43,44]. Others join to learn about alternative treatment options, understand their test results, or to advocate for better awareness of their condition [42,45,46].

As these communities allow varying levels of pseudonymity and anonymity, users with stigmatic and chronic conditions can share intimate or stigmatized information with reduced fear of social repercussions [47,48]. People with chronic conditions often use OHCs to make sense of their experiences and receive validation [49,50].

Studies of OHCs show that an individual member’s support needs as well as the support they provide may change over time and with age [51-53]. Earlier work on support matching suggests that different types of support may be more appropriate for certain needs [54]. A study of a breast cancer OHC found that the presence of emotional or informational support increased the original poster’s satisfaction, though users expressed less satisfaction if they received emotional support when seeking informational support [55]. A separate study on a mental health OHC found that support matching positively predicted satisfaction, but there was significant variance across users [52].

Participating in OHCs empowers members as they become better informed about their health concerns, learn to manage their condition, and gain strategies for communicating with health care providers [22,23,31,43,53,56-58]. In many cases, participants ultimately feel less isolated. Contrary to common belief, Huh [59] found that OHC members do not share misinformation, and they commonly invite peers to consult a provider for medical advice. Other studies of OHCs confirm the beneficial effects of engaging in these communities, showing that members gradually express more positive emotions than negative ones with sustained participation [60,61]. One study of an addiction recovery OHC found that engagement in the community correlates positively with recovery [62].

### Online Endometriosis Communities

Due to the significant impacts of the condition on patients’ lives, people with endometriosis often turn to both offline and online communities for help. The former generally consist of dedicated in-person meetings and activities, and access depends on proximity [63,64]. The latter exist in a variety of forms, such as blogs, mailing lists, Facebook pages, and Instagram accounts; their activities depend on the specific platform [43,49].

Whelan [65] found that both an offline and an online endometriosis group are epistemic communities. As members shared their stories and interacted with peers, they built a new epistemology in which patient experiences became valid forms of knowledge.

Previous research also focuses on the kinds of support and content shared in endometriosis online communities. In a study of Facebook pages for people with endometriosis, Towne et al [11] showed that 48% of the posts provided emotional support, while educational posts made up 21% of the total posts. Furthermore, they found that 94% of the educational posts shared accurate information. By contrast, Metzler et al [10] found that most posts on Facebook and Instagram accounts about endometriosis offered inspiration or support, awareness about the disease, or personal information. Followers mostly engaged with posts that are humorous, generate awareness, and contain personal content. Finally, Shoebotham and Coulson [66] demonstrated that several therapeutic benefits are related to joining endometriosis online support groups. They found that members felt reassured and empowered while improving their knowledge of endometriosis.

## Methods

### Data

Endometriosis OHCs exist on many platforms in many forms [43,49]. We studied *r/Endo* and *r/endometriosis*, two thriving forums about endometriosis on Reddit. Reddit is a social media platform with 93 million daily active users as of September 2024. It is a collection of forums (called subreddits), which users can choose to join, and they may participate as posters or commenters. We focused on Reddit because its OHCs have been thoroughly studied, and previous analyses have demonstrated their potential for understanding patient experiences and improving care [15,17,21]. Furthermore, both *r/Endo* and *r/endometriosis* featured high membership and participation numbers (Table 1) as well showed promise of continued growth (Table 2).

We collected posts and comments from *r/Endo* and *r/endometriosis* from their inception (January 2012 and November 2014, respectively) to December 2021 using the Pushshift Reddit application programming interface (API). The custom Python code used for the data collection process and subsequent analysis are available for reference.

After reading the posts, examining general statistics of the subreddits (Table 1), and comparing their community-specific languages using the “fightin’ words” method by Monroe et al [67], we found that the 2 communities shared sufficient similarities to justify treating them as a single dataset (Multimedia Appendix 1). Members used both subreddits to ask questions about endometriosis, share experiences, and seek advice, and this similarity was reflected in their high vocabulary overlap. Existing differences in word use were relatively minor and not clearly relevant to our RQs.

**Table 1:** General statistics of r/Endo and r/endometriosis subreddits and of the combined dataset.

	r/Endo	r/endometriosis	Combined
Posts (n=34,715), n (%)	22,584 (65.06)	12,131 (34.94)	34,715 (100)
Comments (n=353,162), n (%)	225,221 (63.77)	127,941 (36.23)	353,162 (100)
Members (n=79,004), n (%)	40,734 (51.56)	38,270 (48.44)	79,004 (100)
Active members, n	20,263	17,151	29,783
Unique posters, n	9861	7147	14,910
Words per post, mean (IQR)	184 (76-232)	182 (73-228)	184 (75-230)
Words per comment, mean (IQR)	68 (21-85)	66 (20-83)	67 (20-85)
Comments per post, mean (IQR)	8 (3-10)	9 (3-11)	9 (3-10)
Total number of words (n=29,970,585), n (%)	19,364,282 (64.61)	10,606,303 (35.39)	29,970,585 (100)
Unique tokens, n (%)	90,821	62,757	114,172

**Table 2:** Number of posts and comments in r/Endo and r/endometriosis subreddits over time.

Year	Posts, n	Comments, n
2012	135	1193
2013	0	2978
2014	219	3043
2015	758	6777
2016	1000	6007
2017	1407	13,150
2018	2552	28,335
2019	5041	56,709
2020	9465	104,455
2021	14,138	130,515

### Ethical Considerations

People with endometriosis have historically been failed by research and medical institutions. Similar to other gendered conditions, considering its severity, economic impact, and the number of people diagnosed with it, endometriosis research is greatly underfunded [68]. There is a persistent imbalance between the high percentage of people with endometriosis and the low number of endometriosis experts [2]. Patients deal with ongoing disbelief, invalidation, or trivialization of their symptoms, even from members of the medical community [12,31,33].

Though data from *r/Endo* and *r/endometriosis* are public, members of online communities do not necessarily anticipate that their posts and comments could be used by academic researchers [69]. By collecting, analyzing, and publishing research about these data, we extracted the data from its intended audience and brought it to a new, unanticipated audience [70]. Following previous examples of handling sensitive, health-related data [18,45,71], we obscured the source data to protect members from being identified in relation to their posts or comments. Obfuscation was performed in two ways as follows: (1) throughout this paper, we paraphrased any quoted material and (2) we did not rerelease the underlying text data themselves. Any quoted material in the paper underwent rewording at the sentence level to make it less directly searchable, but we retained as much content of the original version as possible. We released all code and our codebooks so that other researchers may replicate our results on future versions of the OHC, subject to users’ later in situ modifications or deletions of their contributions.

 Because data from Reddit are publicly available, our study was not considered to be human participant research and was exempt from a review by Cornell's institutional review board (IRB0145192). Still, we contacted the moderators of the 2 OHCs to inform them that we were conducting this study.

### Computational Text Analysis

#### Overview

To answer our RQs, we used a combination of natural language processing techniques. These have gained popularity and are being widely implemented in the health care domain, both in research and clinical practice [72,73]. Specifically, we used complementary supervised and unsupervised methods to not only isolate specific instances of personas and intents but also allow topics to emerge beyond the RQs we designed.

#### Topic Modeling

Following suggestions from research on endometriosis experiences [1], we extracted topics from the combined endometriosis OHCs using an abductive approach rather than a priori categories. We used latent Dirichlet allocation (LDA) topic modeling [74], an unsupervised machine learning technique that allows topics to emerge from a corpus of documents without the need to set predefined categories. LDA and similar unsupervised techniques have been successfully used to extract topics from OHCs in previous studies [18,48,75].

LDA identifies groups of similar words (topics) in a corpus based on the statistical probability of these words occurring together. The model assigns, to each document, the likelihood of containing each topic based on the words it features.

Before training the LDA model, we cleaned posts and comments using the string processor included in the little-mallet-wrapper by Antoniak [76], which is designed to prepare raw text for topic modeling. The string processor splits strings into a series of tokens (words separated by punctuation or spaces), removes punctuation and common words, converts all characters to lower case, and returns the transformed string. After this initial cleaning, we removed any post and comment written by or responding to bots by searching for the strings “bot” and “torrent” in both the username and the text of the document. We also removed all documents that had <5 words.

We implemented the LDA function using the *tomotopy* Python package [77]. We experimented by running multiple models with different combinations of the following parameters: number of topics=10;15;20; and 25 and number of most frequently removed words=5;10;15; and 20. We also explored training the model with different document lengths. We first ran LDA on whole posts and comments, and we chunked these into paragraphs and sentences.

To evaluate the performance of each model, we read each topic’s top 100 documents by average probability and assigned a descriptive label to each topic based on the content of those documents and themes previously identified in qualitative research.

Following this evaluation procedure, we found that the topic model trained with 25 topics on paragraph chunks best suited our purposes. We grouped topics into 5 overarching categories based on conceptual similarity and interconnectedness as follows: *symptoms*, *medications*, *health care*, *self-care*, and *life issues*. The detailed description and listing of the 5 categories, the 25 topics, and each topic’s top 10 keywords are shown in the Results section subsequently. We then compared our topics against themes identified in previous research on endometriosis.

#### Supervised Classification

##### Overview

As a complement to the unsupervised topics, we designed 2 supervised tasks as follows: the identification of people based on their social roles (*personas*) in posts and the identification of the goal (*intent*) of a post. Supervised machine learning allowed us to assign OHC-specific labels, including personas and intent, to all posts in our dataset.

##### Personas

Personas are types of people, organized by social roles, who often interact with a person with endometriosis. We identified discussions of personas in endometriosis OHC posts to better understand how endometriosis interfaces with interpersonal relationships. Specifically, we studied the following 4 most frequent personas mentioned in the endometriosis OHCs, based on a qualitative analysis of 200 posts: *medical professionals*, *partners*, *family members*, and *endometriosis OHCs*. Given the variety of terms that could represent each persona (eg, a gynecologist, a subcategory of *medical professional*, could also be referred to as gyno, OB-GYN, gynecologist, obstetrician, doctor, doc, provider, or many others), instead of using a keyword search for each persona category, we trained a supervised model to identify personas based on hand-labeled examples.

Among the personas studied, *medical professional* was any type of professional in the health care system with a patient-facing role, such as a physician, gynecologist, nurse, etc. The *partner* persona included romantic partners, and *family* included mentions of family members (eg, parents, children, and siblings). Depending on paragraph context, *family* also encompassed *partners*. The *endometriosis OHCs* label involved the *r/Endo* and *r/endometriosis* subreddit communities. Paragraphs that mentioned the subreddit did so by name, but they also included posts that spoke directly to the reader (eg, “Can you tell me if you’ve experienced this?”). The *endometriosis OHCs* label differed from the others, given that the endometriosis OHCs tended to be both the audience and subject matter of a post.

In a random sample of paragraphs from posts in the corpus, we assigned the paragraph a label for every present persona category. If there was no persona present, the paragraph did not receive a label. To assess interrater reliability, using the labeling scheme described earlier (alongside a codebook included in Multimedia Appendix 2), 2 authors labeled 200 of the same randomly sampled paragraphs. We then evaluated interrater reliability using Cohen κ. After reaching sufficient interrater reliability (0.7) for all categories, for each persona, one author labeled paragraphs until reaching enough labeled data for acceptable classification performance.

##### Persona Model Setup and Prediction

For each persona category, we fine-tuned a pretrained DistilBERT model on the persona-annotated paragraphs to perform a binary classification task [78]. DistilBERT is an English-language large language model that can be fine-tuned on a given dataset to perform a specific task, such as supervised classification [79]. DistilBERT provides a lightweight version of the BERT encoder language model [79] that retains much of BERT’s performance, making it easier for other works to replicate our results and to use our trained models. For each persona category, we fine-tuned DistilBERT on paragraphs from the combined endometriosis OHCs to best predict the assigned categorical label. We kept all training hyperparameters consistent across models, using a learning rate of 5e-5, 50 warm-up steps, and a weight decay of 0.01 in 3 training epochs. As a baseline model, we also performed logistic regression on each persona category, with input texts in term frequency-inverse document frequency structure. We used each trained model to predict instances of personas in paragraphs in the rest of the corpus. A paragraph could receive more than one persona label, if it mentioned multiple distinct personas.

##### Intent

Previous research on support in OHCs has established multiple overarching categories of support, often characterized as either emotional or informational support [52,53,55,80,81]. OHC research takes these support categories and maps them onto behavioral features in the data, which suggest the type of support a person seeks or provides [51]. Our work specifically considered what users desired from the act of posting, which we call their intent, but we acknowledged that the intent of a post was unavailable to researchers without directly speaking to the person who shared a post. To develop a set of intent categories that are tailored to the endometriosis OHCs, we iteratively labeled, discussed, and revised our labels. We identified 4 common categories of intent as follows: *seeking informational support*, *seeking experiences*, *seeking emotional support*, and *venting*.

##### Seeking Informational Support

Seeking informational support occurred when a person posts on the OHC to find medical information. We built upon previous definitions of seeking informational support [51,82] but incorporated a novel yet simple heuristic for labeling as follows: Could the post’s question be usefully posed to a physician? After revising the *seeking informational support* definition, we found major improvements in labeling consistency, speed, and interrater reliability. Adding this question also created an effective distinction between *seeking informational support* and *seeking experiences*:

My gyno said there’s a chance I have endo, but that I can’t be diagnosed yet since I’m too young (21). Is that true? Is there some sort of test I should be pushing for? I had a doctor who refused to perform a pelvic exam because she said I couldn’t have digestive problems because of endo. I’m feeling skeptical and I don’t know how to advocate for myself.

##### Seeking Experiences

Seeking experiences was the inverse of seeking informational support, as posts that sought experiences could only be answered by someone exposed to the endometriosis experience or who has been on the receiving end of care. Posts that sought experiences asked the community about their experiences with a variety of medical procedures or their day-to-day experiences living with endometriosis. Some of these posts also asked if members of the community had experienced similar symptoms:

Does this sound like endo? How did you get your diagnosis? Did you go to a specialist? Any other advice is appreciated.

##### Seeking Emotional Support

Seeking emotional support included posts that asked for encouragement, empathy, validation, or help navigating emotional situations. These posts might have looked for emotional support after a negative experience, or they might have asked for a celebration from the community after a major milestone in care, such as a diagnosis, improvements in symptoms, or successful self-advocacy:

I’m feeling really down and I can’t talk to my doctor. The only reason she agreed to do this was because of my mental illness. I’m so afraid that either outcome will break my heart. How do I live with the results?

##### Venting

Our final label, venting, occurred when a person posted about their grievances living with endometriosis or frustration at a specific situation. We are not aware of similar labels in previous OHC research. Both communities supported the practice of venting or ranting and even had “flares” (tags) for posts that vent or rant:

This is a long post, but I’m feeling hopeless. I started dealing with things since around 12 years old and now I’m 26. This pain has lasted for weeks and I can’t do any of the physical activities that I love and I feel useless and everyone is dismissing me like a crazy person. I feel dismissed by today’s doctor, some woman on the phone, all the doctors I’ve ever dealt with since 12. Ugh sorry I know this is long but I needed to rant. Anyway thanks for listening to me talk it out.

We found that most of the posts began or ended by stating the person’s intent and their preferred form of support. Whenever possible, we chose the intent that aligned with a post’s explicitly stated purpose.

Using this codebook (Multimedia Appendix 3), one author labeled 1500 sampled posts from *r/Endo* and *r/endometriosis* to be used as training data for our models. Each post could receive between 0 to all 4 intent category labels, though most of the posts had a primary, explicitly expressed intent. A second author labeled 200 of the same posts as those used for training the models to be used for measuring interrater reliability.

##### Intent Models Setup and Prediction

We fine-tuned a series of DistilBERT models on these hand-labeled data to perform binary classification to predict each intent category in a post. We then used the fine-tuned models to predict the intent of posts in the entire corpus. Because there was a binary model fine-tuned to perform classification for each intent category, a post could have 0 to 4 intent classes.

## Results

### RQ1: What Aspects of the Endometriosis Experience Are Discussed in OHCs?

#### Overview

Leveraging topic probabilities, we investigated which aspects of endometriosis experiences were discussed in the endometriosis OHCs. First, we considered what topics emerged from the endometriosis OHCs once we performed LDA topic modeling on paragraph chunks from posts and comments. Second, we analyzed which of those topics were discussed the most in posts.

#### Topics in Posts and Comments

##### Overview

Using LDA topic modeling, we found discussions of 5 main topic categories in the endometriosis OHCs as follows: symptoms, medications, health care, self-care practices, and life issues. A complete list of the 5 qualitatively assigned categories that encompassed our 25 computationally identified topics is provided subsequently. Although health care was the category with the largest number of topics, symptoms and life issues were also commonly discussed in these communities. We further identified a category of self-care practices.

##### Symptoms

A major pattern in the 2 OHCs was the presence of topics related to symptoms (Table 3). People with endometriosis experience a wide range of chronic symptoms such as *gastrointestinal* issues; *pelvic floor* pain; heavy, irregular, and painful *menstruation*; *muscular* cramps in their legs and abdomen; and others. Many users shared these symptoms with the communities in the hope of receiving or providing support.

**Table 3:** Topics in the symptoms category. Topic numbers were assigned randomly by the model, while labels were assigned upon reading the top 100 documents for each topic.

Topic number	Label	Top 10 words
0	Gastrointestinal	Take, nausea, bowel, stomach, help, water, constipation, taking, drink, helps
3	Pelvic floor	Pelvic, floor, therapy, physical, help, sex, helped, therapist, muscles, lot
5	Menstruation	Period, periods, days, bleeding, symptoms, painful, heavy, cramps, started, normal
17	Muscular	Back, sex, right, feel, feels, lower, side, sometimes, left, painful
21	Sharing symptoms	Feel, period, day, days, bad, time, every, worse, back, last

##### Medications

Due to the chronic nature and current incurability of endometriosis, people with endometriosis use a variety of drugs and treatments. Users of the 2 OHCs often listed their *pain management* routine, shared *hormonal treatment experiences* (“18 months ago I started using the Nuva ring and I love it”), recounted the side effects of specific drugs they have used, or provided medical *information on hormonal drugs* (“Orlissa is a GnRH antagonist, so it lowers estrogen directly without relying on the same feedback mechanism as Lupron”). The *medications* category grouped these experiences (Table 4).

**Table 4:** Topics in the medications category. Topic numbers were assigned randomly by the model, while labels were assigned upon reading the top 100 documents for each topic.

Topic number	Label	Top 10 words
14	Pain management	Work, take, cbd, time, day, job, days, help, use, much
18	Hormonal drug experiences	Control, months, birth, iud, pill, period, years, mirena, periods, got
23	Drugs	Take, side, taking, effects, weight, months, pill, medication, dose, NUMmg
24	Information on hormonal drugs	Control, birth, symptoms, treatment, side, estrogen, effects, hormones, lupron, hormonal

##### Health Care

In the *health care* category, we grouped topics regarding the medical aspects of endometriosis and how patients with endometriosis experienced the health care system (Table 5). Often, senior members of the OHCs provided new users with *medical information* on the condition, overviews on the process of *getting diagnosed*, as well as *information on surgery*. Users also advised each other on how to prepare for their *medical appointments*. They often pointed to competent endometriosis *specialists*, compared *insurance* policies, and highlighted helpful *online resources*.

**Table 5:** Topics in the health care category. Topic numbers were assigned randomly by the model, while labels were assigned upon reading the top 100 documents for each topic.

Topic number	Label	Top 10 words
1	Information on surgery	Lap, excision, weeks, first, back, still, time, two, months, post
2	Medical information	Cyst, ovary, uterus, endometriosis, cysts, removed, tissue, ovaries, ultrasound, found
4	Getting diagnosed	Symptoms, blood, could, ultrasound, bladder, test, issues, tests, doctor, endometriosis
6	Online resources	Nook, endometriosis, https, nancy, group, //www, research, Facebook, list, doctors
9	Specialists	Doctor, specialist, find, see, excision, doctors, one, endometriosis, good, need
13	Insurance	Insurance, medical, health, hospital, work, care, pay, need, doctor, live
20	Medical appointments	Doctor, going, ask, see, thank, anyone, appointment, sure, want, think

##### Self-Care

As endometriosis requires a considerable amount of *self-care* (Table 6), patients were faced with the challenge of caring for themselves while maintaining other responsibilities. Users of the OHCs found support against exhaustion and isolation by comparing experiences and tips about their *postsurgery recovery*. They also provided detailed information on their *diet*, *product recommendations* for items that help with daily activities, and various *comfort items* for when symptoms flared up.

**Table 6:** Topics in the self-care category. Topic numbers were assigned randomly by the model, while labels were assigned upon reading the top 100 documents for each topic.

Topic number	Label	Top 10 words
8	Postsurgery recovery	Day, days, first, home, time, around, back, gas, hours, week
15	Product recommendations	Heating, pad, use, hot, heat, one, water, help, helps, pads
19	Diet	Diet, eat, gluten, food, foods, dairy, eating, try, free, lot
22	Comfort items	Wear, pants, belly, look, weight, one, size, wearing, super, cup

##### Life Issues

The last category, *life issues*, included groups users’ discussions of general life issues connected with having a severe chronic condition (Table 7). In these communities, users shared experiences of *dismissal and abuse* and their *medical stories* as patients. They gave each other support through their *fertility* struggles. Community members exchanged expressions of *gratitude* and *empathy* with their peers.

**Table 7:** Table 7. Topics in the life issues category. Topic numbers were assigned randomly by the model, while labels were assigned upon reading the top 100 documents for each topic.

Topic number	Label	Top 10 words
7	Dismissal	People, even, doctors, think, feel, something, say, one, want, women
10	Gratitude	Hope, thank, good, much, sorry, better, feel, luck, well, find
11	Medical stories	Years, told, doctor, said, got, went, diagnosed, back, lap, finally
12	Fertility	Pregnant, want, kids, years, hysterectomy, fertility, pregnancy
16	Empathy	Feel, people, life, want, help, much, need, support, sorry, hard

#### Most Discussed Topics in Posts

**Figure 1:**
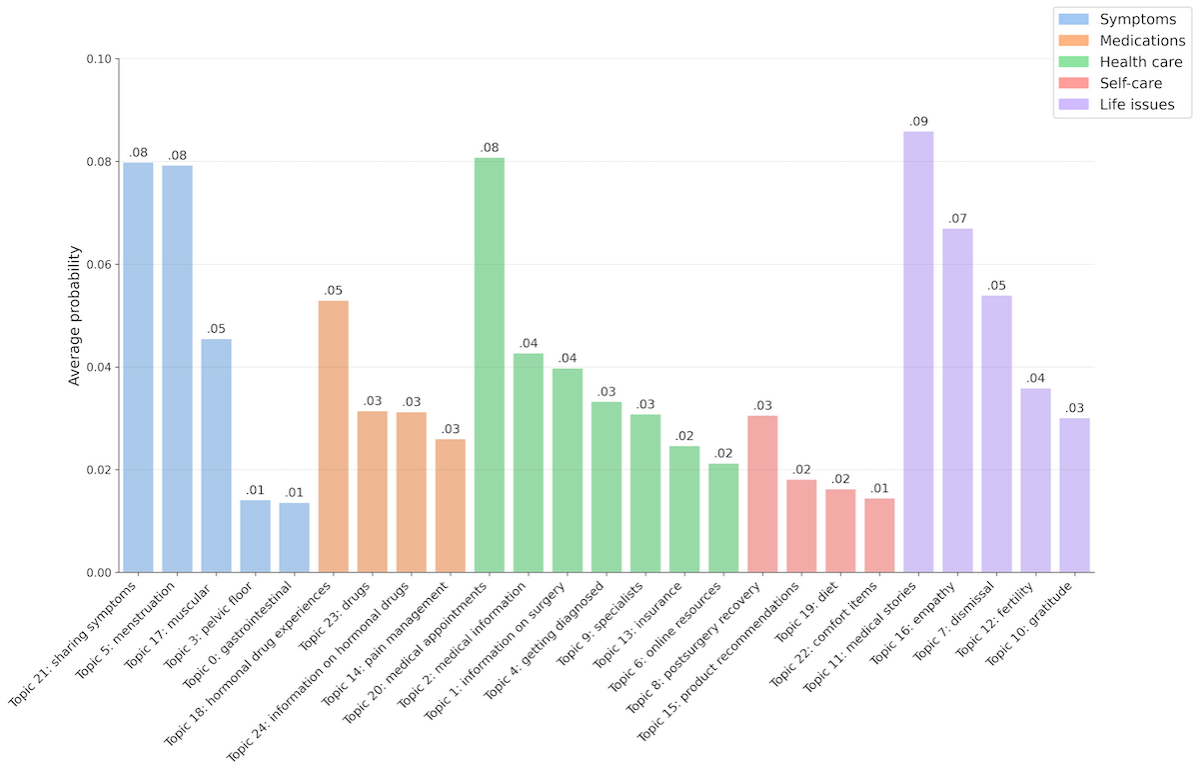
Average topic probabilities in posts collected from the 2 endometriosis online health communities ordered by category. Medical stories, medical appointments, sharing symptoms, menstruation, and empathy had the highest average probability.

To investigate which aspects of the experiences of patients with endometriosis were most discussed in the endometriosis OHCs, we measured which topics had the highest average probability in all posts. If a topic had a high average probability across all posts, it indicated that the topic was highly present in the endometriosis OHCs. In posts, the topics with the highest average probability were *medical stories*, *medical appointments*, *sharing symptoms*, *menstruation,* and *empathy* (Table 8, Figure 1).

We found that *medical stories* and *medical appointments* were the 2 most discussed topics. New or returning users frequently recounted their health care journey at the beginning of their posts—from their first symptoms as teenagers to undergoing surgery and choosing between treatment options. Users also asked specific questions, such as how to book their medical appointment, what to do if an appointment was moved or the physician did not show up, and what strategies others used to communicate successfully with their physicians.

Two *symptom* topics, *sharing symptoms* and *menstruation*, were among the most frequent topics. Users of the endometriosis OHCs shared detailed accounts of their symptoms in order to elicit their peers’ opinions on whether they should seek urgent care, whether a new symptom might be caused by their treatment rather than by endometriosis, and whether what they were going through resembled other people’s experiences with endometriosis.

**Table 8:** Topics with the highest average probability in posts.

Topic number	Label	Probability, mean
11	Medical stories	0.086
20	Medical appointments	0.081
21	Sharing symptoms	0.080
5	Menstruation	0.079
16	Empathy	0.067

A large number of posts in the OHCs were solely dedicated to describing menstrual symptoms. New users of these communities were often undiagnosed teenagers who wondered whether they should seek medical assistance given their experiences with menstruation. Furthermore, endometriosis was typically treated with hormonal medicines, which caused additional changes to patients’ menstrual cycles. Patients shared such changes with peers to understand if the treatment had been effective at relieving their pain.

*Empathy* was the fifth most present topic in posts of the 2 OHCs, underlining that demonstrations of empathy were valued by patients with endometriosis. Users often also lamented feeling misunderstood and dismissed.

### RQ2: What Aggregate Needs Emerge From the OHCs?

#### Overview

In this section, we consider the needs expressed by members of the OHCs. For each of the topics outlined in RQ1, we considered the following: (1) Which topics are more likely when different personas are mentioned? and (2) What is the intent of posts when they mention each topic? By doing so, we could better understand the interplay between endometriosis experiences, interpersonal relationships, and the goals of OHC members.

#### Persona Model Validation

Before analyzing personas in the OHCs, we validated the model by considering interrater reliability in our hand-labeled dataset and the classification performance of our model across the different classes and assessed the balance between false positives and negatives for each persona.

First, as shown in Table 9, we reached sufficient interrater reliability for each of the persona classes. The lowest agreement occurred for paragraphs that were assigned the family label and the highest for paragraphs assigned to the medical professional label.

Next, we considered classification performance for each of the fine-tuned persona models.

Classification accuracy for a holdout test set of 25% of the total labeled paragraphs is listed in Table 10. For all classification results, we presented macroaveraged scores, which is a more pessimistic scoring method that treats both classes equally, regardless of class imbalance. All 4 fine-tuned models reached a high classification performance.

**Figure 2:**
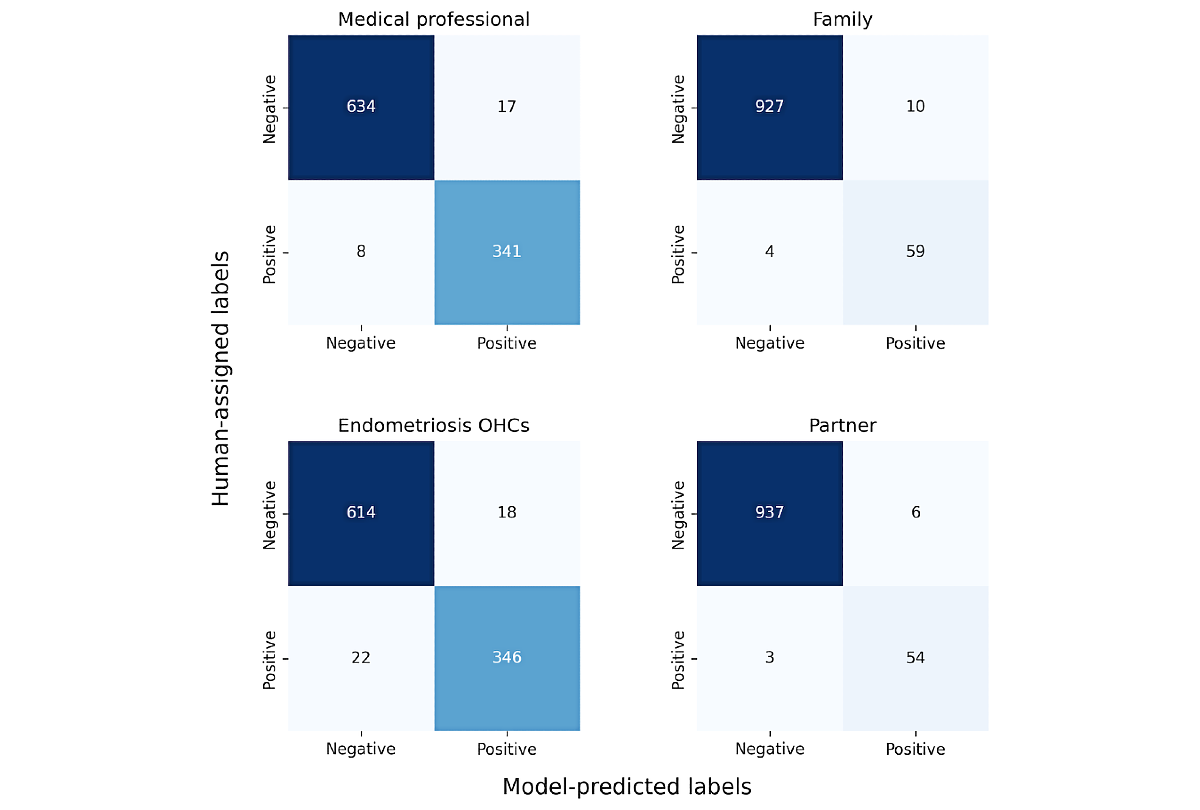
Confusion matrices for the 4 persona categories. Positive refers to the presence of the persona category in the paragraph. OHC: online health community.

In addition to the high classification performance listed in Table 10, we considered the frequency of false positives and negatives for each class through confusion matrices (Figure 2). Each confusion matrix compared the hand-labeled persona categories with the model-predicted categories for all annotated paragraphs. On the basis of these confusion matrices, we found that each model performed quite well, but false positives were more likely for physician, family, and partner labels and false negatives for the endometriosis OHC when the model was incorrect.

**Table 9:** Number of paragraphs assigned to the persona labels of the total paragraphs labeled and interrater reliability.

Persona	Paragraphs assigned to labels, n (%)	Interrater reliability (Cohen κ; 200 post subset)
Family (n=1500)	153 (10.2)	0.79
Partner (n=2000)	166 (8.3)	0.83
Medical professional (n=1000)	349 (34.9)	0.87
Endometriosis online health communities (n=1000)	368 (36.8)	0.84

**Table 10:** Classification performance for each persona category for both logistic regression and DistilBERT. All scores are reported as macroaverages.

Persona and classifier		Precision	Recall	*F*_1_-score
**Family**				
	Logistic regression	0.50	0.45	0.48
	DistilBERT	0.94	0.92	0.93
**Partner**				
	Logistic regression	0.50	0.46	0.48
	DistilBERT	0.91	0.97	0.93
**Medical professional**				
	Logistic regression	0.71	0.83	0.72
	DistilBERT	0.93	0.93	0.93
**Endometriosis online health communities**				
	Logistic regression	0.72	0.83	0.73
	DistilBERT	0.93	0.92	0.92

#### Personas in the OHCs

Of the 4 persona categories, posts on the endometriosis OHC most often mentioned the *endometriosis OHCs*, followed by *medical professional*, *family*, and *partners* (Table 11).

**Figure 3:**
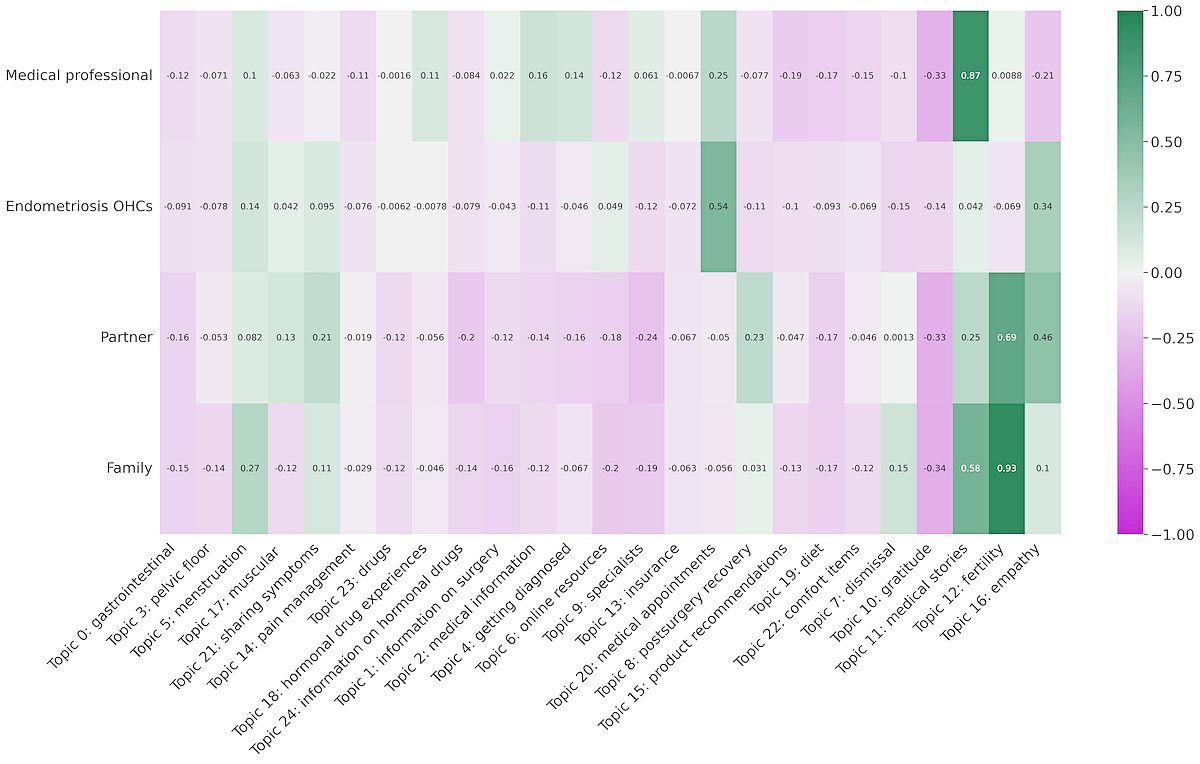
Average topic probabilities (converted to z scores) for posts with different personas. OHC: online health community.

Of the posts predicted with at least 1 of the 4 personas, we found the topics that were most present. For each persona, we found the average topic probabilities for all posts predicted with each persona converted to *z* scores. Figure 3 displays this result, depicting the members of the OHCs who were most likely to discuss when they mentioned each persona.

When a *medical professional* was mentioned, posts were more likely about *medical appointments* and *medical stories*, highlighting the important role that providers had in shaping patient medical pathways. However, *medical professional* was the least likely of any persona to be discussed in combination with *empathy* (*P*<.001).

Interestingly, posts in the *endometriosis OHCs* were more likely to discuss *medical appointments* than posts in *medical professional* (*P*<.001). In alignment with our findings in RQ1, users of the OHC requested the assistance of the community to prepare for visits, as this support might not have been available to them in clinical settings.

Posts that mentioned *partner* or *family* were likely to discuss topics from the *life issues* category, in particular *fertility* (*P*<.001). These posts often emphasized how navigating fertility deeply affected relationships. Mentions of *family* in posts about *fertility* might have to do with family planning and personal goals in growing a family. Some users expressed concern about being able to have or keep a partner when dealing with infertility. These posts also mentioned feeling pressured to have children by family or partners.

Finally, posts that mentioned *partner* often also discussed *postsurgery recovery* (*P*<.001). Partners can indeed play an important role in helping patients with endometriosis access treatment and maintain self-care routines. In addition, it was sometimes the partner of a person with endometriosis who asked for advice from the OHC.

**Table 11:** Percentage of posts with more than one mention of each persona in the endometriosis online health communities (OHCs; n=34,715).

Persona	Posts with >1 mention, n (%)
Medical professional	20,187 (58.15)
Endometriosis OHCs	25,650 (73.89)
Partner	4113 (11.85)
Family	4968 (14.3)

#### Intent Model Validation

As with our persona models, we first validated our intent models by assessing interrater reliability, classification performance, and the presence of false positives and negatives.

We reached acceptable interrater reliability across all categories (Table 12). The interrater reliability for intent was quite similar to the scores for personas; highest agreement was reached for posts that sought experiences and the lowest agreement for posts that included venting.

Classification accuracy for the fine-tuned intent models on a held-out test set of 25% of the total labeled paragraphs is listed in Table 13. Overall, the intent models reached acceptable performance, though this performance was lower than that of our persona models. This slightly lower performance was expected because of the more complex nature of the intent categories.

**Figure 4:**
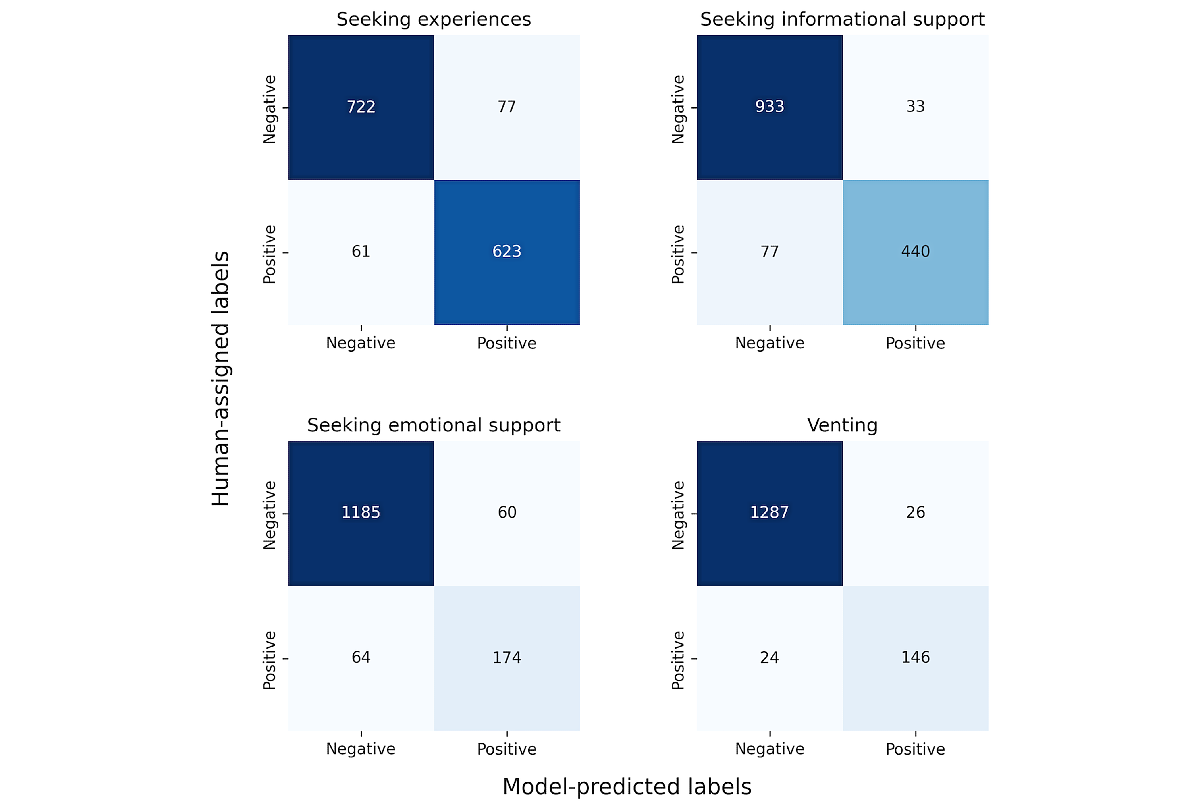
Confusion matrices for the 4 intent categories. Positive refers to the presence of the intent category in the post.

We then considered the balance between false positives and negatives for the intent models. Figure 4 shows the confusion matrices comparing the ground-truth intent labels with the model predictions for data where the ground-truth intent was not none. We found that when the model was incorrect, false positives and negatives were nearly equally likely for venting and seeking emotional support intents, false positives were more likely for seeking experiences intent, and false negatives were more likely for seeking informational support intent.

**Table 12:** Number of posts assigned to the intent labels and interrater reliability for each label (n=1500).

Label	Posts assigned to the label, n (%)	Interrater reliability (Cohen κ; 200 post subset)
Seeking informational support	524 (34.93)	0.79
Seeking experiences	691 (46.07)	0.83
Seeking emotional support	241 (16.07)	0.76
Venting	172 (11.47)	0.74

**Table 13:** Classification performance for each intent category for both logistic regression and DistilBERT. All scores are reported as macroaverages.

Intent and classifier		Precision	Recall	*F*_1_-score
**Seeking informational support**				
	Logistic regression	0.59	0.71	0.56
	DistilBERT	0.86	0.82	0.84
**Seeking experiences**				
	Logistic regression	0.75	0.75	0.75
	DistilBERT	0.83	0.83	0.83
**Seeking emotional support**				
	Logistic regression	0.51	0.80	0.47
	DistilBERT	0.72	0.69	0.70
**Venting**				
	Logistic regression	0.50	0.44	0.47
	DistilBERT	0.83	0.80	0.81

#### Intents in the OHCs

We considered the goals of members of the community in their posts through our algorithmic intent predictions. Across all posts, we found that users were most likely to *seek experiences* from the OHC; they did so in roughly half of the posts. *Seeking informational support* occurred in around a quarter of the posts, and *seeking emotional support* and *venting* were the least common intent types (Table 14).

We found answers to the following questions: (1) When a member sought information from the community, what were they trying to learn about? And (2) When a member simply wanted to vent, what subjects were most often related to their frustration?

**Figure 5:**
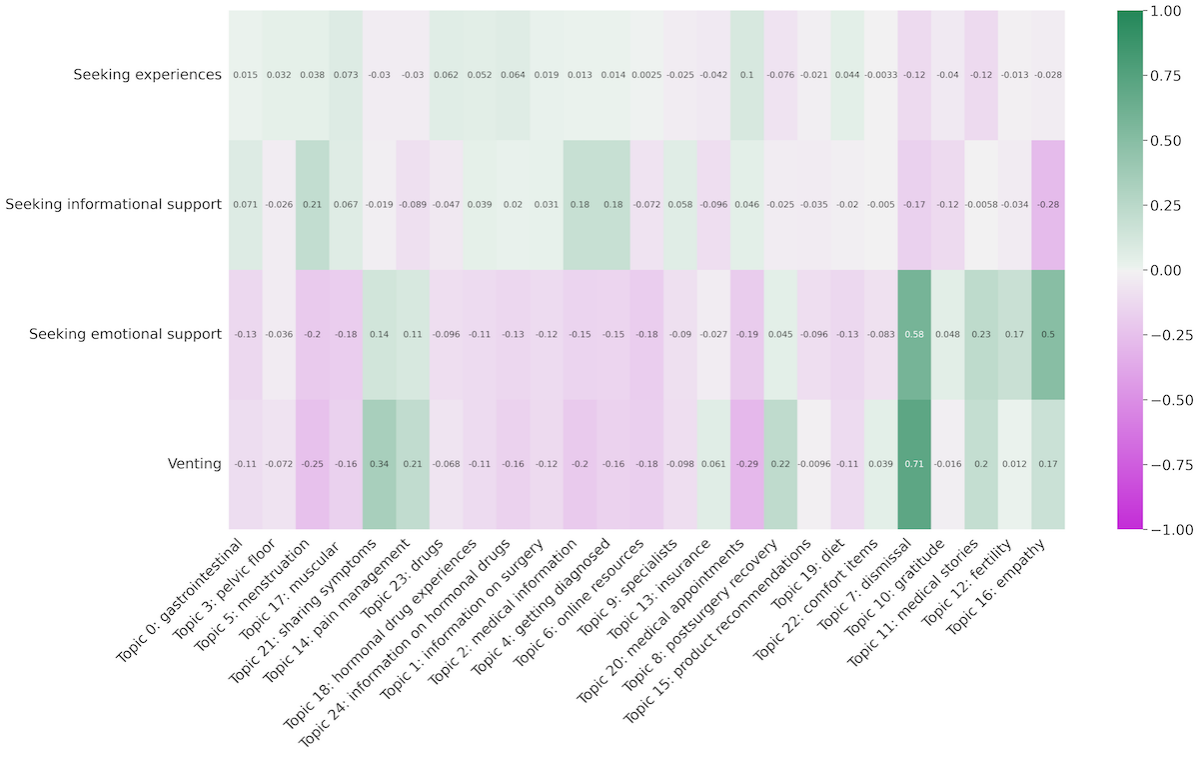
Average topic probabilities (converted to z scores) for posts with different intents.

We found an important divide between the subject matter of posts that *sought experiences* or *informational support* and those that *sought emotional support* or *vented* (Figure 5). The subject matter of posts that *sought information* or *experiences* was more often about topics in the *symptoms*, *medications*, and *health care* categories, while members were more likely to *seek emotional support* and *vent* about the *life issues* topics, including *dismissal*, *medical stories*, *fertility*, and *empathy*.

However, members of the endometriosis OHCs did *seek emotional support*, and *vented*, about *pain management* and when *sharing symptoms*. While a person with endometriosis might look for information or experiences regarding their symptoms and pain, they were more likely to look for emotional support from the community or to vent their frustrations.

**Table 14:** Percentage of posts with each intent label in the endometriosis online health communities (n=34,715).

Persona	Posts with >1 mention (%)
Seeking informational support	9696 (27.93)
Seeking experiences	17,060 (49.14)
Seeking emotional support	5374 (15.48)
Venting	3457 (9.96)

## Discussion

### RQ1: What Aspects of the Endometriosis Experience Are Discussed in OHCs?

Using topic modeling we find that OHCs are spaces dedicated to narrations of users’ health care pathways, directions on how to find care and manage symptoms, as well as expressions of validation between peers regarding their health concerns.

Particularly, the most discussed topics in the 2 communities are *medical stories*, *medical appointments*, *sharing symptoms*, *menstruation*, and *empathy*. These results extend previous findings from small-scale, qualitative research collecting experiences of patients with endometriosis. These include evidence of the benefits of sharing one’s story within a community [49,65], the need for assistance with treatment regimens and appointments [14,31], the uncertainty experienced by patients related to their symptomatology [13], as well as the value of receiving validation regarding health concerns and symptoms [8].

An existing study of a PCOS subreddit has also found concordance between the OHC user population and research-identified patient cohorts [17]. Although the PCOS OHC includes patients that are typically excluded from clinical trials (such as those with multiple conditions), trends found in laboratory test results posted to the community are consistent with clinically reported results.

Our results also align with studies on OHCs, showing that OHC users become better at communicating with their providers and managing their conditions as well as feel less isolated [23,43,49,50,56-58].

### RQ2: Which Aggregate Unmet Needs Emerge From the OHCs?

We find that posts mention the endometriosis OHCs more than they mention medical professionals, highlighting the vital role that these groups play in the users’ health care decisions, and most of the posts are written to seek experiential advice. Venting is the least common of our intent categories, but venting still occurs in a substantial fraction (10%) of the posts.

We find that users need assistance with accessing and preparing for medical visits as well as navigating fertility options. To meet these needs, patients currently turn to the OHCs, their partners, and their family. Interestingly, members of the OHCs seldom associate medical professionals and providers with empathy.

We also find that patients’ relationships with their partners and family members can be affected by their condition. Users share how physical manifestations of endometriosis, such as infertility, alter their life goals and complicate personal relationships. At the same time, partners and family members play a vital role by serving as informal caregivers. They even use the OHCs for advice in creating a strong support system.

Furthermore, while users seek experiential knowledge regarding treatments and health care processes, they also wish to vent and establish an emotional connection about the life-altering aspects of the condition.

These results align with previous research on the areas of endometriosis care that need improvement, including nonholistic treatments [1-3], unsatisfactory patient–health care provider communication [5,9,18], and the lack of training or educational resources for patients’ loved ones [1,7-9].

### Recommendations for Clinical Practice

The potential implications for clinical practice of this work are significant. Physicians who treat endometriosis would benefit from knowing the depth and breadth of medical information shared on these forums. Patients belonging to such communities often have a higher baseline level of knowledge about endometriosis and will benefit from more nuanced discussion of symptoms and treatment options. In addition, if physicians are aware of the overall supportive nature of these communities, they may be able to recommend them to patients who feel isolated with their diagnosis.

On a wider policy level, this work highlights the need for better education about endometriosis in medical schools and obstetrics and gynecology residency training programs. With improved awareness of the condition, it is less likely that communications with patients would be perceived as dismissive. In addition, this work calls attention to the need for more physicians with expertise in endometriosis, as highlighted by the popularity of posts on finding endometriosis specialists.

### Limitations

Limitations of the study include its sole focus on Reddit, which may exclude patients who prefer receiving support on other social media platforms or through offline groups. Our focus on 2 primarily English-language subreddits may not capture the experience of non–English-speaking users.

In addition, we cannot assume that every post describes endometriosis, as many users rely on the community for information before an official endometriosis diagnosis. While this provides us with valuable information about the prediagnosis experience, it is possible that users post, in some instances, about medical issues that are not endometriosis.

### Future Work

The study spans nearly a decade, but it does not explicitly analyze how trends in discussion topics or user needs evolve over time (eg, the period before the COVID-19 pandemic vs the period after the COVID-19 pandemic). Future work could perform temporal analyses designed to detect how external factors, such as a pandemic or major developments in endometriosis research, change discussion topics and user needs in the community.

In addition, while we learn vital information about the endometriosis experience, there is a more complex picture of how endometriosis is impacted by social conditions. Future work could analyze how social determinants (eg, employment status and cultural stigma) influence the endometriosis experience.

### Conclusions

In this study, we conducted a large-scale analysis of user needs in 2 endometriosis OHCs, *r/Endo* and *r/endometriosis*. We found that these communities provide members with a space where they can discuss care pathways, learn to manage symptoms, and receive validation. Our results point to the need for greater empathy within clinical settings, easier access to appointments, more information on health care processes, and further support for patients’ loved ones.

Our study demonstrates the value of quantitative analyses of OHCs. OHCs provide very large datasets on patient experiences. In this work, we analyzed hundreds of thousands of posts and comments by tens of thousands of users. To the best of our knowledge, the sample size of this study is one order of magnitude larger than the sample size of any other study on the needs and experiences of patients with endometriosis. Therefore, our results reinforce and extend findings from small-scale studies about patient experiences and provide insights into hard-to-reach groups. Finally, we believe that studies of OHCs can help design interventions to improve care, as argued in previous studies [15,17,18,45].

## Appendix

Multimedia Appendix 1 Fightin’ Words results.

Multimedia Appendix 2 Persona codebook.

Multimedia Appendix 3 Intent codebook.

## Abbreviations

LDA: latent Dirichlet allocation
OHC: online health community
PCOS: polycystic ovary syndrome
RQ: research question

## Data Availability

The datasets generated and analyzed during this study are not publicly available to protect the privacy of the members of the online health communities studied in this work but are available to researchers by the corresponding author on reasonable request. The code and related materials necessary to reproduce the results of this study are available [83].
