# Supplementary material for: Endometriosis Communities on Reddit: Quantitative Analysis
Source: J Med Internet Res. 2025 Mar 31;27:e57987. doi: 10.2196/57987 (PMC11997530; doi:10.2196/57987)
Supplement: Multimedia Appendix 1 [file jmir_v27i1e57987_app1.docx]

## Multimedia Appendix 1 - Fightin’ Words Results

We implement Fightin’ Words [1] to examine similarities and differences between the vocabularies of r/Endo and r/endometriosis. As represented in Table 1, the two communities have a number of words that distinguish them from each other, suggesting that there may be some differences in norms. However, the words that vary most between the communities (e.g. short or lap) do not relate directly to our project’s research questions and hypotheses, though it is surprising to see some words, including symptoms, distinguish the communities.

Table 1. Fightin’ words scores comparing r/Endo (left) to r/endometriosis (right).

| **r/Endo** | | **r/endometriosis** | |
| --- | --- | --- | --- |
| **Word** | **z-score** | **Word** | **z-score** |
| lap | 49.64 | short | -30.58 |
| she | 44.56 | pick | -27.48 |
| still | 39.16 | ways | -26.26 |
| didnt | 39.1 | via | -25.67 |
| better | 38.46 | themselves | -25.61 |
| see | 36.29 | system | -25.52 |
| work | 35.7 | wife | -25.43 |
| excision | 35.7 | become | -25.34 |
| find | 35.42 | wanna | -25.28 |
| we | 35.31 | welcome | -24.64 |
| since | 34.66 | head | -24.37 |
| symptoms | 34.49 | entire | -24.04 |
| might | 34.48 | diagnostic | -23.94 |
| people | 34.41 | diarrhea | -23.8 |
| sure | 34.34 | caffeine | -23.72 |
| right | 34.21 | thankfully | -23.66 |
| need | 33.7 | date | -23.49 |
| lot | 33.53 | whether | -23.37 |
| got | 33.28 | besides | -23.33 |
| youre | 33.09 | seemed | -23.19 |
| make | 32.69 | round | -23.19 |
| things | 32.5 | beginning | -23.17 |
| said | 32.19 | gotten | -22.67 |
| specialist | 31.87 | therapy | -22.39 |
| cant | 31.55 | laps | -22.23 |

In addition, we perform FightinWords on each subreddit against r/PCOS to control for possible errors. We choose r/PCOS since it’s an OHC dedicated to discussing a similar severe chronic female genital condition, polycystic ovary syndrome. Interestingly, the similarity between r/Endo and r/endometriosis vocabularies is further supported in this step. As shown in the table below, there is great overlap in the top terms of r/Endo and the top terms of r/endometriosis when compared to r/PCOS. Results are displayed in Tables 2 and 3. In consideration of our hypotheses and the scope of this project, we find that the two subreddits share consistent enough similarities to allow for merging them into a single dataset.

Table 2. Fightin’ words scores comparing r/Endo (left) to r/pcos (right).

| **r/Endo** | | **r/pcos** | |
| --- | --- | --- | --- |
| **Word** | **z-score** | **Word** | **z-score** |
| pain | 162.26 | pcos | -100.47 |
| endo | 116.64 | weight | -79.57 |
| surgery | 90.12 | hair | -72.01 |
| endometriosis | 66.79 | diet | -48.89 |
| pelvic | 49.07 | acne | -48.36 |
| he | 45.65 | loss | -44.31 |
| specialist | 44.77 | lose | -41.51 |
| lap | 42.17 | low | -38.87 |
| they | 41.23 | eating | -38.67 |
| bowel | 39.28 | high | -38.5 |
| uterus | 38.81 | level | -36.87 |
| cramp | 38.54 | test | -36.41 |
| painful | 37.38 | eat | -36.22 |
| during | 37.34 | taking | -34.76 |
| laparoscopy | 35.82 | sugar | -34.52 |

Table 3. Fightin’ words scores comparing r/endometriosis (left) to r/pcos (right).

| **r/endometriosis** | | **r/pcos** | |
| --- | --- | --- | --- |
| **Word** | **z-score** | **Word** | **z-score** |
| pain | 156.76 | pcos | -75 |
| endo | 105.61 | weight | -58.85 |
| surgery | 86.37 | hair | -49.72 |
| endometriosis | 79.02 | diet | -36.18 |
| pelvic | 44.59 | acne | -35.22 |
| cramp | 40.6 | loss | -32.63 |
| painful | 39.11 | lose | -30.42 |
| sex | 38.44 | low | -28.93 |
| they | 38.26 | level | -28.68 |
| bowel | 38.19 | high | -28.64 |
| lap | 37.83 | eating | -28.52 |
| laparoscopy | 37.04 | eat | -27.88 |
| during | 36.36 | taking | -26.9 |
| he | 35.4 | sugar | -25.51 |
| uterus | 34.9 | skin | -25.37 |

## References

1. Monroe BL, Colaresi MP, Quinn KM. Fightin’ Words: Lexical Feature Selection and Evaluation for Identifying the Content of Political Conflict. Polit Anal 2008;16(4):372–403. doi: 10.1093/pan/mpn018
