# Supplementary material for: Endometriosis Communities on Reddit: Quantitative Analysis
Source: J Med Internet Res. 2025 Mar 31;27:e57987. doi: 10.2196/57987 (PMC11997530; doi:10.2196/57987)
Supplement: Multimedia Appendix 2 [file jmir_v27i1e57987_app2.docx]

## Multimedia Appendix 2 - Persona Codebook

B.1 General instructions:

- Label each paragraph based on whether or not the type of persona is mentioned.
- These can be discussions of actual experiences with the given persona, or hypothetical discussions about interacting with a person.

B.2 Medical Professionals

- Includes people who are employed by a medical institution in a patient-facing role.
- Common types of medical professionals include gynecologists, endometriosis specialists, nurses, therapists, etc.

B.3 Family

- Includes anyone in a familial role in position to the person with endometriosis.
- We do not restrict family to immediate family members.
- Partners may be included in this label if they are described as long-term partners. Choose the best option based on context.
- Common types of family members include mothers, fathers, siblings, significant others, children, cousins, aunts, uncles, grandparents, etc.
- At times, people will discuss wanting to have children. Since we include hypothetical discussions of personas, we would include hypothetical children within the family persona.

B.4 Partners

- Includes people who are in a romantic relationship with the person with endometriosis.
- Common types of partners include husbands, wives, SOs, girlfriends, boyfriends, partners, etc.

B.5 Endometriosis Online health Communities

- Includes mentions of the community itself.
- If the post is directed toward the community as an audience and mentions the community while posing questions (e.g. "have you all..."), we include this as a mention of the endometriosis OHC.
- Common mentions of the endometriosis OHC use the terms reddit, subreddit, r/Endo or r/endometriosis, endo warriors, or general phrases like "this community" or "on here"
- Does not include general mentions of a community of people with endometriosis; must specifically be people in the online community.
- For this label especially, consider post context when determining if the endometriosis OHC is or is not present.
