# Supplementary material for: Endometriosis Communities on Reddit: Quantitative Analysis
Source: J Med Internet Res. 2025 Mar 31;27:e57987. doi: 10.2196/57987 (PMC11997530; doi:10.2196/57987)
Supplement: Multimedia Appendix 3 [file jmir_v27i1e57987_app3.docx]

## Multimedia Appendix 3 - Intent Codebook

C.1 General instructions:

- Many posts explicitly restate their intent at the bottom of the post (and sometimes in the introduction) – whenever possible, use the explicitly stated intent
- Many posts have to share medical information or histories as a way of getting to their intent. In this case, the intent isn’t necessarily about sharing medical information
- Posts should typically have one to two intents
  - Some examples:
    - A post may ask multiple questions: if one is informational and the other is for experiences, that would be 2 intents
    - A post may mostly vent but also periodically ask for information, that would also be two intents
- The hardest part will probably be separating seeking information from experiences & separating seeking emotional support from venting

C.2 Seeking Informational Support

- Asking for fact-based information that could just as easily be asked to a medical professional
  - Helpful heuristic: could someone ask their doctor this question?
- Asking about medical details, whether a set of symptoms sounds like endometriosis, etc.

C.3 Seeking Experiences

- In ways, this is the inverse to “seeking informational support”→these are the questions you can’t ask doctors
- Asking for descriptions about experiences with surgery, diagnosis, treatment, medicine
- Asking for advice based on personal experiences (what has/hasn’t worked for others)
- Asking whether other people have experienced something (“has anyone had X symptom”)

C.4 Seeking Emotional Support

- Explicitly looking for comfort or peace of mind
- Sharing positive news and asking for people to celebrate with them
- Posting to the community because no one else in their life “understands”
- Asking for advice about dealing with emotional problems in their relationships, how to not be anxious about their condition/surgery, etc.
- This label has a wider range of positive to negative emotions
- *Note: for this label, look for explicit requests for comfort/peace of mind. Don’t project “seeking emotional support” just because someone is frustrated–they might be frustrated and just want medical information*
